# Clustering in Large Networks Does Not Promote Upstream Reciprocity

**DOI:** 10.1371/journal.pone.0025190

**Published:** 2011-10-05

**Authors:** Naoki Masuda

**Affiliations:** 1 Department of Mathematical Informatics, The University of Tokyo, Bunkyo, Tokyo, Japan; 2 PRESTO, Japan Science and Technology Agency, Kawaguchi, Saitama, Japan; Umeå University, Sweden

## Abstract

Upstream reciprocity (also called generalized reciprocity) is a putative mechanism for cooperation in social dilemma situations with which players help others when they are helped by somebody else. It is a type of indirect reciprocity. Although upstream reciprocity is often observed in experiments, most theories suggest that it is operative only when players form short cycles such as triangles, implying a small population size, or when it is combined with other mechanisms that promote cooperation on their own. An expectation is that real social networks, which are known to be full of triangles and other short cycles, may accommodate upstream reciprocity. In this study, I extend the upstream reciprocity game proposed for a directed cycle by Boyd and Richerson to the case of general networks. The model is not evolutionary and concerns the conditions under which the unanimity of cooperative players is a Nash equilibrium. I show that an abundance of triangles or other short cycles in a network does little to promote upstream reciprocity. Cooperation is less likely for a larger population size even if triangles are abundant in the network. In addition, in contrast to the results for evolutionary social dilemma games on networks, scale-free networks lead to less cooperation than networks with a homogeneous degree distribution.

## Introduction

Several mechanisms govern cooperation among individuals in social dilemma situations such as the prisoner's dilemma game. Upstream reciprocity, also called generalized reciprocity, is one such mechanism in which players help others when they themselves are helped by other players. It is a form of indirect reciprocity, in which individuals are helped by unknown others and vice versa [1], [2].

Cooperation based on upstream reciprocity has been observed in various laboratory experiments. Examples include human subjects in variants of the trust game, which is a social dilemma game [3]–[5], human subjects participating in filling out tedious surveys [6], and rats pulling a lever to deliver food to a conspecific [7]. Even more experimental evidence is available in the field of sociology in the context of social exchange [8], [9] (also see [10], [11] for classical examples of the Kula ring).

Nevertheless, theory and numerical simulations have revealed that upstream reciprocity in isolation does not promote cooperation (but see Barta et al. [12] for an exception). Upstream reciprocity usually supports cooperation only when combined with another mechanism that can yield cooperation on its own. Cooperation appears when the population size is small [13], [14], upstream reciprocity is combined with direct reciprocity or spatial reciprocity [15], players move across groups [16], players interact assortatively [17], or players inhabit heterogeneous networks [18].

**Figure 1:**
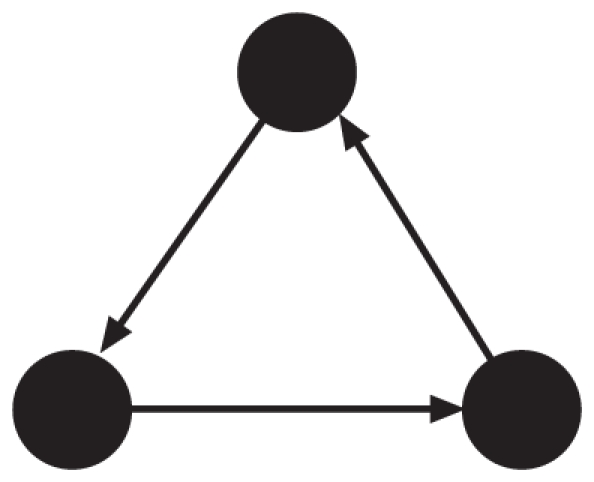
Directed cycle with 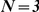 nodes.

In their seminal study, Boyd and Richerson analyzed an upstream reciprocity game on a directed cycle and showed that it yields cooperation only when the cycle is small [13]. The shortest possible cycle with indirect reciprocity consists of three players ( Fig. 1) because a cycle composed of two players only involves direct reciprocity. Cooperation is intuitively less likely for longer cycles because a player that helps a unique downstream neighbor on the cycle has to “trust” too many intermediary players for their tendency to cooperate before the player eventually receives help.

Real social networks are full of short cycles represented by triangles, a feature known as transitivity [19] or clustering [20]–[22]. Therefore, a natural expectation is that larger networks with a high level of clustering (i.e., many triangles) may facilitate cooperation based on upstream reciprocity [8]. In the present study, I address this issue theoretically. I extend the model of Boyd and Richerson [13] to general networks and derive the condition under which the unanimity of players using upstream reciprocity is resistant to invasion of defectors. Then, I apply the condition to model networks to show that clustering does little to promote cooperation except in an unrealistic network. This conclusion holds true for both homogeneous and heterogeneous networks, where heterogeneity concerns that in the degree, i.e., the number of neighbors for a player.

My results seem to contradict previous results for spatial reciprocity in which clustering enhances cooperation in the prisoner's dilemma game [23] and those for heterogeneous networks in which heterogeneity enhances cooperation in various two-person social dilemma games [24]–[27] and in the upstream reciprocity game [18]. These previous models are evolutionary, however, whereas mine and the original model by Boyd and Richerson [13] are nonevolutionary and based on the Nash equilibrium. I opted to use a nonevolutionary setting in this study because interpretation of evolutionary games seems elusive for heterogeneous networks [28], [29] (see Discussion for a more detailed explanation).

## Results

### Preliminary: upstream reciprocity on a directed cycle

Boyd and Richerson proposed a model of upstream reciprocity on the directed cycle [13]. By analyzing the stability of a unanimous population of cooperative players, they showed that cooperation is unlikely unless the number of players, denoted by 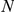, is small.

In their model, the players are involved in a type of donation game. Each player may donate to a unique downstream neighbor on a directed cycle at time 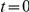 by paying cost 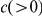. The recipient of the donation gains benefit 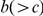. Among the recipients of the donation at 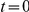, those who comply with upstream reciprocity donate to a unique downstream neighbor at 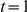 by paying cost 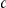. Chains of donation are then carried over to downstream players, who may donate to their downstream neighbors at 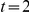. At 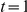, defectors that have received a donation at 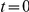 terminate the chain of donation. Such defectors receive benefit 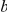 at 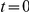 and lose nothing at 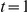. This procedure is repeated for all players until all the chains of donation terminate. If all the players perfectly comply with upstream reciprocity, the chains never terminate. In contrast, if there is at least one defector, all the chains terminate in finite time.

As in iterated games [30], [31], 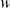 (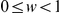) is the probability that the next time step occurs. We can also interpret 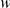 as the probability that players complying with upstream reciprocity do donate to their downstream neighbors, such that they erroneously defect with probability 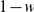 in each time step. Each player's payoff is defined as the discounted sum of the payoff over the time horizon. In other words, the payoff obtained at time 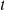 (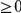) contributes to the summed payoff with weight 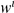.

It may be advantageous for a player not to donate to the downstream neighbor to gain benefit 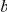 without paying cost 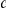 over the time course. However, a player that complies with upstream reciprocity may enjoy a large summed payoff if chains of donation persist in the network for a long time.

Each player is assumed to be of either classical defector (CD; termed unconditional defection in [13]) or generous cooperator (GC; termed upstream tit-for-tat in [13]). By definition, a CD does not donate to the downstream neighbor at 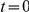 and refuses to relay the chain of donation received from the upstream neighbor to the downstream neighbor at 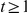. A GC donates at 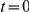 and donates to the downstream neighbor if the GC received a donation from the upstream neighbor in the previous time step.

For this model, Boyd and Richerson obtained the condition under which the unanimity of GCs is robust against the invasion of a CD (i.e., conversion of one GC into CD). When all players are GC, the summed payoff to one GC is equal to
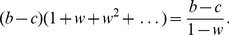
(1)If 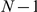 players are GC and one player is CD, the unique CD's summed payoff is given by
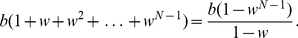
(2)Therefore, GC is stable against the invasion of CD if the right-hand side of Eq. (1) is larger than that of Eq. (2), that is,
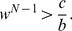
(3)Equation (3) generalizes the result for direct reciprocity [30], [31], which corresponds to the case where 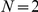. Equation (3) also implies that cooperation is likely if 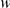 is large. However, maintaining cooperation is increasingly difficult as 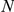 increases.

### Model

I generalize the Boyd-Richerson model on a directed cycle to the case of general networks. Consider a network of 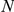 players in which links may be directed or weighted. I denote the weight of the link from player 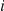 to 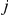 by 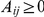. I assume that the network is strongly connected, i.e., any player is reacheable from any other player along directed links. Otherwise, chains of donation starting from some playes never return to them because of the purely structural reason. In such a network, it would be more difficult to maintain cooperation than in strongly connected networks. Even for strongly connected networks that might accommodate upstream reciprocity, I will show that cooperation is not likely for realistic network structure.

Assume that all the players are GC and that each GC starts a chain of donation of unit size at 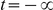. Therefore, the total amount of donation flowing in the network in each time step is equal to 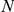. In the steady state, the total amount of donation that each player receives from upstream neighbors is equal to that each player gives to downstream neighbors in each time step. I denote the total amount of donation that reaches and leaves player 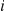 by 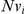, where 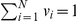. In this situation, the amount of donation that player 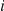 imparts to player 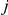 in each time step is equal to 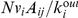, where 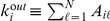 is the outdegree of player 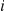. Player 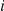 receives payoff 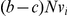 in each time step.

In our previous work [18], we assumed that each GC starts a unit flow of donation at 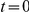. In the present study, however, I wait until the flow reaches the steady state before starting the game at 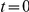.

The definition of CD for general networks is straightforward; a CD donates to nobody for 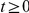. I extend the concept of GC to the case of general networks as follows. On a directed cycle, a GC quits helping its downstream neighbor once the GC is not helped by the upstream neighbor [13]. On a general network, the total amount of donation that GC 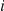 receives per unit time in the absence of a CD is equal to 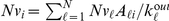. If there is a CD, the total amount of donation that GC 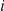 receives may be smaller than the amount that player 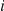 would receive in the absence of a CD. By definition, the GC responds to this situation by relaying the total amount of the incoming donation proportionally to all its downstream neighbors in accordance with the weights of the links outgoing from player 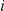.

As an example, suppose that one upstream neighbor of GC 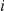, denoted by 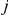, is CD and all the other 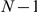 players, including player 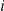, are GC. At 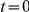, the total amount of donation that 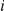 receives is equal to 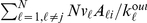, which is smaller than 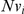. Player 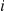 donates 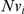 in total. Therefore, player 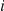
's payoff at 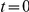 is equal to 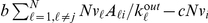. In response to the amount of donation that player 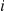 received at 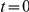, player 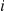 adjusts the total amount of donation that it gives the downstream neighbors from 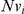 to 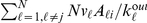 at 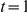. Therefore, player 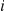 donates 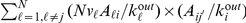 to its downstream neighbor 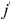. This quantity is smaller than the donation that player 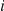 would give player 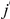 in the absence of CD 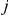, which would be equal to 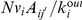.

An implicit assumption is that the GC cannot identify the incoming links along which less donation is received as compared to the case without a CD. In other words, even if a GC is defected by the CD in the upstream, the GC cannot directly retaliate. Instead, the GC distributes the retaliation equally (i.e., proportionally to the weight of the link) to its downstream neighbors.

### Stability of upstream reciprocity in networks

In this section, I derive the condition under which no player is motivated to convert from GC to CD when all the players are initially GC.

The steady state 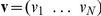 is equivalent to the stationary density of the simple random walk in discrete time. It is given as the solution of
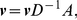
(4)where 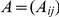 is the 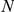-by-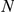 adjacency matrix, where 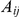 represents the weight of the link from 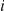 to 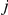, and the diagonal matrix 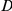 is defined as 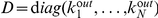. The 
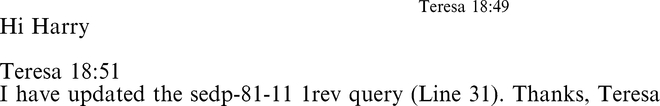
 element of 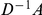 is equal to 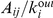, that is, the probability that a walker at node 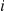 transits to node 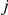 in one time step. If the network is undirected, the solution of Eq. (4) is given by 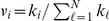, where 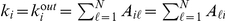.

The summed payoff to player 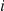 is equal to
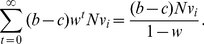
(5)


To examine the Nash stability of the unanimity of GC, I analyze the situation in which player 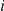 is CD and the other 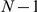 players are GC. At 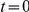, the 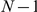 GCs pay 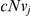 (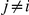), and player 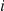 pays nothing. Therefore, the benefits to the 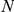 players, including player 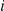, at 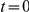 are given in vector form by
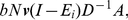
(6)where 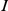 is the 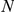-by-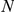 identity matrix, and 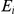 is the 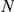-by-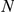 matrix whose 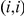 element is equal to one and all the other elements are equal to zero. The benefit to player 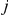 (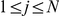) at 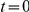 is equal to the 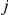
th element of the row vector given by Eq. (6).

At 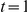, the downstream neighbors of player 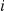 donate less because player 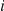 defects at 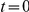. The amount of donation given to player 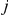, where 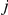 is not necessarily a neighbor of 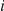, at 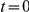 is equal to the 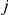
th element of the row vector 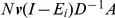. Therefore, the total amount that GC 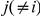 donates to its downstream neighbors at 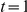 is equal to the 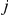
th element of 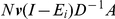. Player 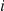, who is CD, does not donate to others at 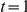. Therefore, the amount of the donation issued by the players at 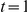 is represented in vector form as 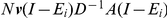. The discounted benefits that the players receive at 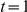 are given in vector form by
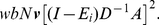
(7)By repeating the same procedure, we can obtain the summed benefits to the players in vector form as
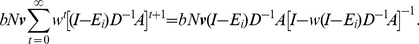
(8)To derive Eq. (8), I used the fact that the spectral radius of 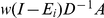 is smaller than unity (that of 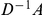 is equal to unity). The 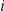
th element of Eq. (8) is equal to the summed payoff to player 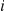 because player 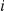 does not pay the cost to donate at any 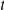.

If the 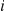
th element of Eq. (8) is smaller than the quantity given by Eq. (5), player 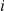 is not motivated to turn from GC to CD. Therefore, the unanimity of GC is stable if and only if
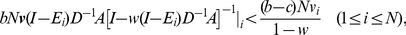
(9)where 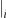 indicates the 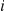
th element of a vector. By rearranging terms of Eq. (9), I obtain
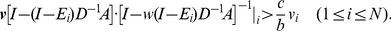
(10)Because 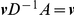, Eq. (10) can be reduced to
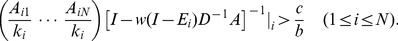
(11)


Equation (11) is never satisfied when 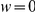 because 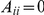. It is always satisfied when 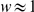 because the left-hand side of Eq. (10) tends to 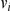 as 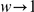.

For a directed cycle having 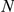 nodes, 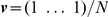, 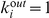 (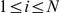), and 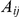 is equal to 1 if 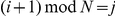 and 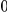 otherwise. Owing to the symmetry with respect to 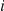, we only have to consider the condition (i.e., Eq. (9) or Eq. (11)) for player 1 and obtain the following:
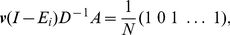
(12)

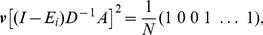
(13)

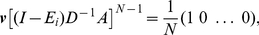
(14)

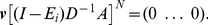
(15)Therefore, Eq. (11) can be read as 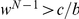, which reproduces the result by Boyd and Richerson [13].

### Numerical results for various networks

For general networks, calculating 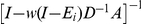, which is used in Eqs. (9) and (11), is technically difficult because this matrix may have nondiagonal Jordan blocks. Standard formulae for decomposing matrices under independence of different eigenmodes do not simply apply. The method for efficiently calculating 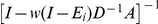 is described in the Methods section.

I conducted numerical simulations for different networks to determine the threshold value of 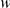, denoted by 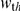, above which the unanimity of GC is stable against invasion of CD. The conclusions derived from the following numerical simulations are summarized as follows: (a) abundance of triangles (and other short cycles) hardly promotes cooperation, and (b) networks with heterogeneous degree distributions yield less cooperation than those with homogeneous degree distributions.

#### Network models

I use five types of undirected networks generated from four network models. It would be even more difficult to obtain cooperation in directed networks because undirected networks generally allow more direct reciprocity than directed networks (see Discussion for a more detailed explanation).

The regular random graph (RRG) is defined as a completely randomly wired network under the restriction that all nodes (i.e., players) have the same degree 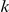 [21], [22]. The RRG has low clustering (i.e., low triangle density) and is homogeneous in degree [21], [22], [32].

To construct a network from the Watts-Strogatz (WS) model [32], nodes are placed in a circle and connected such that each one is adjacent to the 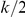 closest nodes on each side on the circle. In this way, each node has degree 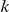. A fraction 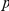 of the links is then rewired, and a selected link preserves one of its end nodes and abandons the other end node. Then, I randomly select a node from the network as the new destination of the rewired link such that self-loops and multiple links are avoided. I use two cases, one in which 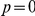 and the other in which 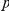 is small but greater than zero. In both cases, the network has a high amount of clustering. When 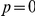, the network is homogeneous in degree and unrealistic because it has a large average distance between nodes. When 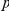 is positive and appropriately small, the degree is narrowly distributed and the network has a small average distance [32].

As an example of networks with heterogeneous degree distribution, I use the Barabási-Albert (BA) model. It has a power-law (scale-free) degree distribution 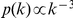, a small average distance, and low level of clustering [20], [33].

To probe the effect of triangles in scale-free networks, I use a variant of the Klemm-Eguluz (KE) model [34], [35]. For appropriate parameter values, my variant of the KE model generates scale-free networks with 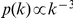, small average distances, and a high level of clustering.

#### The effect of clustering

For a fixed network and a fixed value of cost-to-benefit ratio 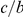, the threshold value of 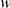 above which the unanimity of GC is stable against conversion of player 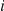 into CD depends on 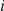. I denote this value by 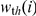. I determine 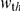 as the largest value of 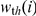 (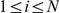). This is true because once a certain player 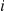 turns from GC to CD, other players may be also inclined to turn to CD. It is straightforward to extend the condition shown in Eq. (9) to the case of multiple CD players. For example, we can similarly derive the condition under which player 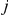 turns from GC to CD when player 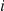 (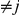) is CD and all the other 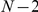 players are GC. For example, on the left-hand side of Eq. (9), we just need to replace 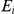 with 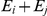. I confirmed for all the following numerical results that once a player turns from GC to CD, some others are also elicited to turn from GC to CD according to the Nash criterion and that such a transition from GC to CD cascades until all players are CD. In loose terms, this phenomenon is reminiscent of models of cascading failure of overloaded networks, which mimic, for example, blackouts on power grids [36].

**Figure 2:**
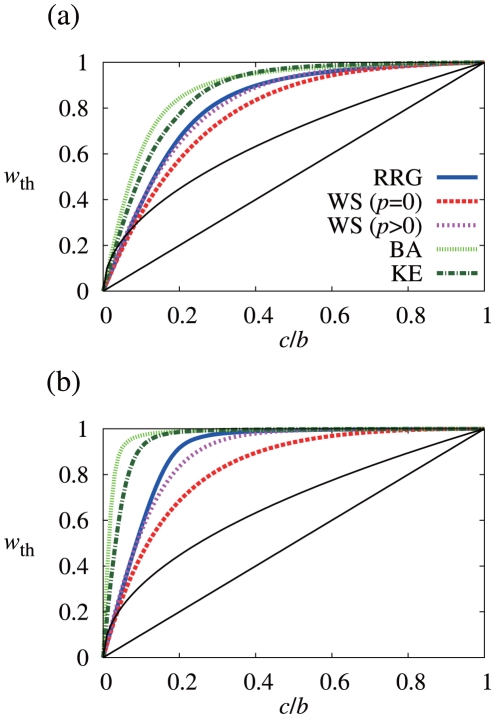
Relationship between threshold discount factor (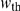) and cost-to-benefit ratio (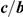). I use the five types of networks and set (a) 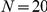, 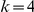, and (b) 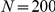, 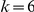. The results for direct reciprocity (i.e., 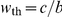) and upstream reciprocity on the directed triangle (i.e., 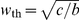) are also shown by thin black lines for comparison. In (a), I set the rewiring probability for the WS model to 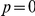 and 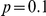. For the BA model, there are initially 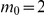 nodes (i.e., dyad), and the number of links that each added node has is set to 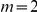. For my variant of the KE model, the initial number of nodes and the number of links that each added node has are set to 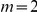, and an active node 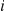 is deactivated with probability proportional to 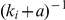, where 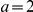. After constructing the network based on the original KE model [34], I rewire fraction 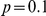 of randomly selected links to make the average distance small. In (b), I set 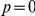 and 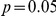 for the WS model, 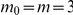 for the BA model, and 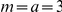 and 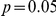 for the KE model.

The relationship between 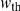 and 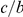 is shown in Fig. 2(a) for the five networks with 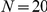 and mean degree 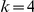. The parameter values for the networks are explained in the caption of Fig. 2. A small 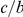 value results in a small 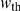 value, indicating that cooperation is facilitated. This is generally the case for various mechanisms for cooperation [2], [37].

For reference, the results for direct reciprocity (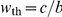) and upstream reciprocity on the directed triangle (Fig. 1; 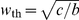) are also shown in Fig. 2(a) by thin black lines. Except for small 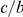 values, the five networks with 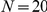 possess higher 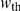 values as compared to these reference cases.

The two networks generated from the WS model yield smaller values of 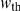 than those obtained from the RRG, indicating that the WS model allows more cooperation than the RRG. Because the degree distributions of these networks are almost the same and the average distances of the RRG and the WS model with 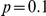 do not differ by much [32], I ascribe this difference to clustering. An abundance of triangles and short cycles in networks (i.e., the WS model) enhances cooperation. However, the difference in 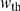 is not very large. In quantitative terms, clustering does little to promote cooperation.

The same conclusion is supported for heterogeneous networks (the BA and KE models). Values of 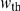 for the KE model, which yields a high level of clustering are smaller than those for the BA model, which yields a low level of clustering. However, the 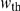 values for the KE model are considerably larger than those for the RRG and the WS model, and the differences between the results for the BA and KE models are small.

**Figure 3:**
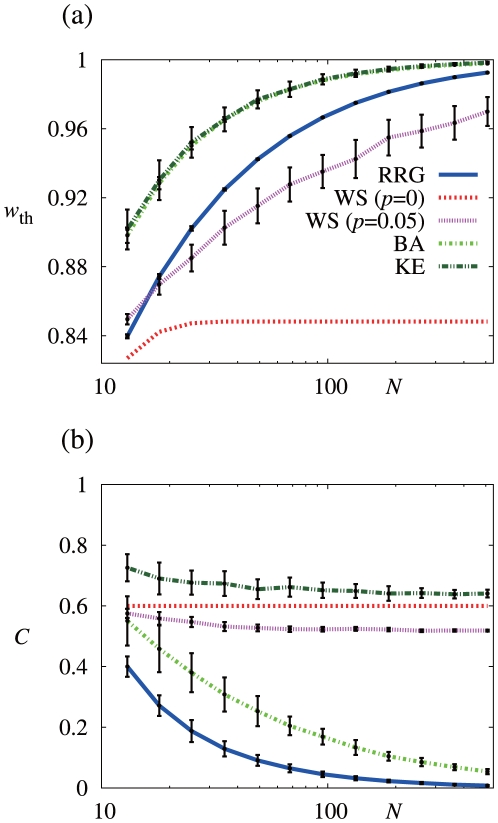
Effects of network size (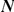). (a) Dependence of the threshold discount factor (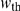) on 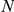. (b) Dependence of the clustering coefficient (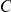) on 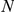. I use the five types of networks and set 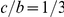. The parameter values for the networks are the same as those used in Fig. 2(b). In (a), the results for the BA and KE models heavily overlap.

To summarize, clustering promotes cooperation but only to a small extent. To further substantiate this finding, I looked at different cases. Figure 2(b) compares 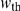 and 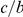 values for the networks with 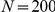 and 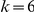. Figure 3(a) shows the dependence of 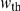 on 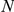 when 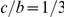. These cases also suggest that clustering hardly promotes cooperation.

#### Scale-free versus homogeneous networks

Figure 2 indicates that scale-free networks (i.e., the BA and KE models) allow less cooperation than networks with a homogeneous degree distribution (i.e., the RRG and WS model). This is in contrast with the results for the evolutionary two-person social dilemma games [24]–[27] and those for the evolutionary upstream reciprocity game [18] on heterogeneous networks in which scale-free networks promote cooperation. The difference stems from the fact that players in evolutionary games mimic successful neighbors, whereas in my Nash equilibrium model, players judge whether GC or CD is more profitable when the other players do not change the strategies (see Discussion for a more detailed explanation).

**Figure 4:**
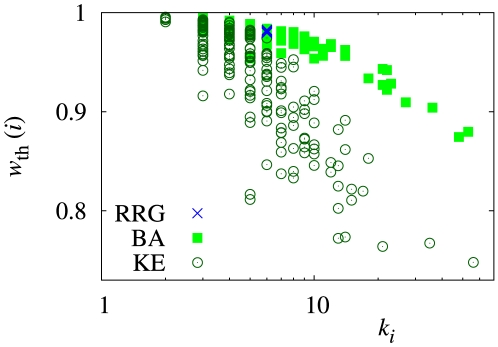
Relationship between threshold discount factor (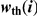) and node degree (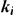). I use the RRG, the BA model, and the KE model with 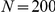 and 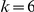, and set 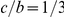. The parameter values for the networks are the same as those used in Fig. 2(b).


To probe the reason why cooperation is reduced on scale-free networks, I examine the dependence of the player-wise threshold value, i.e., 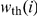 for player 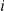, on node degree 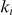. I generate a single network from each of the RRG, the BA model, and the KE model with 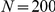 using the same parameter values as those used in Fig. 2(b). For 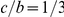, the relationship between 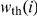 and 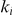 is shown in Fig. 4 for all nodes in the three networks. 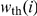 decreases with 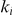 in the BA and KE models. In the RRG, 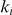 is equal to 6 for all the nodes, and the value of 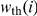 is approximately the same for all the nodes.

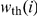
 and 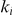 are negatively correlated because the amount of donation flow that a putative CD 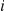 stops is strongly correlated with 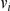. At 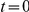, it is equal to 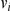. At 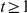, it is generally smaller than 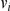, but player 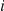 having a large 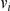 value tends to receive a large inflow of donation, which player 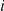 stops in the next time step. For undirected networks, 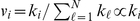 holds true. Players with small degrees are therefore tempted to convert to CD because the impact of the player's behavior (i.e., to donate or not to donate) on the entire network is small. Therefore, a small 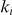 leads to a large 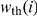. Even for directed networks, 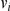 and 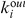 are often strongly correlated [38]–[40]. Because the minimum degree in a scale-free network is smaller than that in a homogeneous network if the mean degree of the two networks is equal, scale-free networks have larger 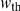 as compared to homogeneous networks.

#### Cooperation in large networks

A comparison of Figs. 2(a) and 2(b) suggests that a large 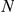 makes cooperation unlikely. To examine this point further, I set 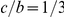, generated 100 networks for each 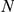 value and each network type, calculated 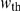, and obtained the mean and the standard deviation of 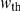. Because the WS model with 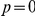 is unique for a given 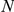, the mean and standard deviation are not relevant in this network.

The mean and standard deviation of 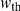 for the five networks of various sizes are shown in Fig. 3(a). The results for the BA and KE models heavily overlap. Cooperation is less likely as 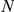 increases in all models, except for the WS model with 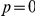. This result is consistent with that for a directed cycle [13].


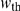
 increases with 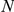 not entirely owing to the decreased level of clustering in the network. To show this, I plot the mean and standard deviation of the clustering coefficient 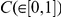, which quantifies the abundance of triangles in a network [32], in Fig. 3(b). The clustering coefficient is defined as 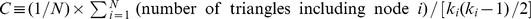. Figure 3(b) indicates that 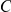 decreases with 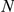 for the RRG and the BA model. Therefore, the effect of 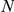 and 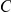 on 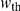 may be mixed in these two network models. However, 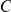 stays almost constant for the WS and KE models. At least for these models, an increase in 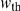 is considered to originate primarily from an increase in 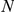, not from changes in the level of clustering.

In Fig. 3(a), 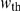 seems to approach unity as 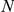 increases except for the WS model with 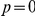. As previously stated, the WS model with 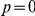 is unrealistic because it has a large average distance between pairs of nodes [20]–[22], [32]. Therefore, I conclude that cooperation based on upstream reciprocity is not likely for homogeneous and heterogeneous networks in general.

## Discussion

I generalized the upstream reciprocity model proposed for a directed cycle [13] to general networks and reached two primary conclusions.

First, cooperation based on upstream reciprocity is not likely in general networks regardless of the abundance of triangles and heterogeneity in the node degree. Because the networks that I examined are undirected, some amount of direct reciprocity is relevant; GC neighbors partially retaliate directly against a CD. My result that cooperation is unlikely for undirected networks implies that cooperation would be even more difficult for directed networks in which direct reciprocity is less available. In directed networks, direct reciprocity occurs only on reciprocal links between a pair of players.

Second, I showed that scale-free network models allow less cooperation (i.e., large 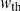) as compared to networks with homogeneous degree distributions. This result is opposite of those for two-person social dilemma games [24]–[27] and the upstream reciprocity game [18]. The difference stems from the fact that the previous studies assumed evolutionary games and the present study (and the original model by Boyd and Richerson [13]) is based on nonevolutionary analysis.

I adopted a nonevolutionary setup and examined the condition for the Nash equilibrium because the concept of the evolutionary game on heterogeneous networks seems elusive. Evolutionary games on heterogeneous networks imply that a player imitates the strategy of a successful neighbor that is likely to have a different node degree. However, players with different degrees are involved in essentially different games because the number of times that each player plays the game per generation necessarily depends on the degree. Therefore, for example, a small-degree player cannot generally expect a large payoff by mimicking a successful neighbor with a large degree. In this situation, defining the game and payoff for players with various degrees is complicated [26], [28], [29]. Use of the Nash criterion does not incur this type of problem.

The overall conclusions of the present study are negative. To explain the occurrence of upstream reciprocity in real societies, it may be advantageous to combine upstream reciprocity with other non-network mechanisms, such as the ones mentioned in the Introduction.

## Methods

### Numerical methods for calculating Eqs. (9) and (11)

I determined 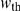 by applying the bisection method to Eq. (9) or (11). To calculate 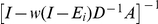 for different values of 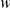, it is beneficial to use the expansion of 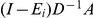 in terms of independent modes. This is possible when the adjacency matrix 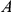 for the subnetwork composed of the GCs is diagonalizable, as shown below.

I assume that there are 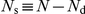 GCs and 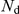 CDs. In the main text, I focused on the case 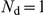. However, the case 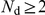 is also relevant because I verified in the main text that the appearance of a single CD leads to the further emergence of CDs. Without loss of generality, I assume that players 1, 2, …, 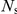 are GC and players 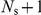, 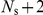, 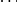, 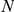 are CD, and that the network is strongly connected. We need to identify all the (generalized) eigenmodes of 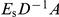, where
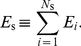
(16)I first partition 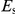, 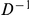, and 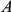 into two-by-two blocks, each partition corresponding to the set of GC and that of CD. For a candidate of a left eigenvector of 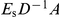, denoted by 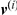,
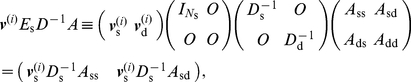
(17)where 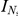 is the identity matrix of size 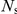; 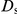 and 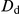 are diagonal matrices whose diagonal entries are equal to the outdegrees of the GCs and CDs, respectively; 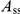 is the 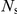-by-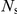 matrix corresponding to the adjacent matrix within the GCs; and 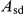, 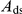, and 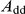 are similarly defined blocks of the original adjacency matrix 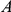. Note that 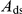 and 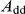 are absent on the right-hand side of Eq. (17) and as such are not relevant to the following discussion.

First of all, 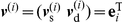 (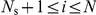) is a trivial zero left eigenvector of 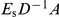. Here, 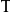 denotes transpose, and 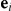 is the unit column vector in which the 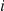
th element is equal to unity and all the other elements are equal to zero.

To obtain the other 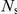 generalized eigenmodes of 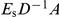, I consider the case in which 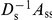 is diagonalizable. Otherwise, efficiently calculating 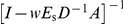 via matrix decomposition is difficult. 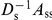 is diagonalizable if the network is undirected. A diagonalizable 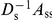 possesses 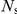 nondegenerate left eigenvector 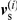 (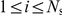) with the corresponding eigenvalue 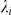. It is possible that 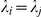 for 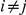.

If 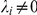, 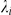 is an eigenvalue of 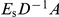, and the corresponding left eigenvector is given by 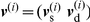, where
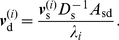
(18)


If 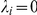, Eq. (17) implies that 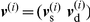 is not a left eigenvector of 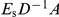. An example network with 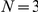 that has nontrivial zero eigenvalues is presented in the next section for a pedagogical purpose. When 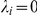, I set 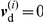 such that
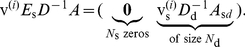
(19)Because 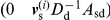 can be represented as a linear sum of 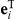 (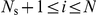), 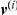 is a type of generalized eigenvector corresponding to 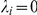.

I denote by 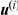 (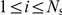) the nontrivial generalized right eigenmodes of 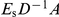 corresponding to 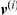. To obtain 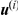, I denote by 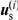 (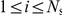) the normalized right eigenvectors of 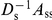 with eigenvalue 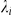. Then,
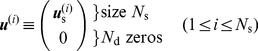
(20)are right eigenvectors of 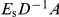 that respect the orthogonality 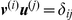, where 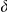 is the Kronecker delta.

For completeness, I obtain the expression of the other 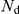 right eigenvectors of 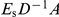 corresponding to the trivial zero eigenvalue as follows. I align 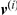 and 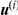 (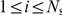) such that nonzero eigenvectors correspond to 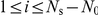 and generalized zero eigenmodes correspond to 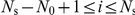. Then, the orthogonality condition 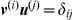 reads
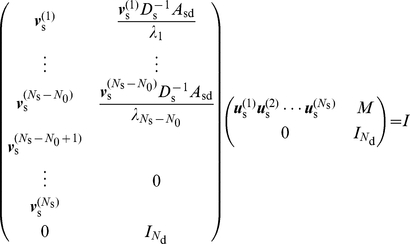
(21)for an 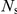-by-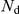 matrix 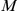. Equation (21) yields
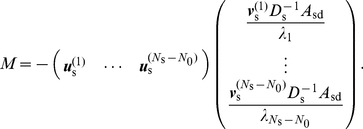
(22)


Finally, the decomposition of 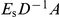 is given by
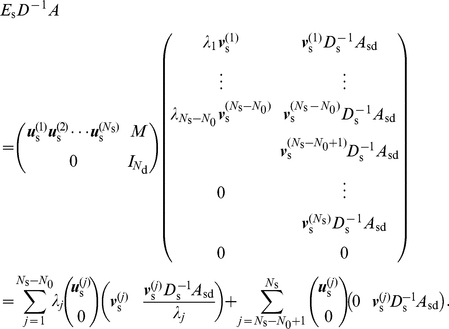
(23)Combining Eq. (23) and the orthogonality condition 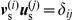, I obtain
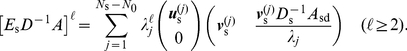
(24)


Using Eqs. (16), (23), and (24), we can express the quantities appearing on the left-hand sides of Eqs. (9) and (11) as
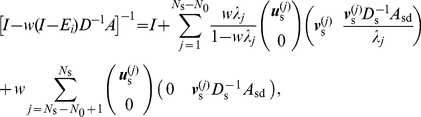
(25)

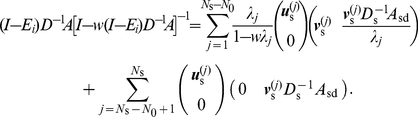
(26)


If 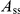 is symmetric, 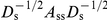 is also symmetric and therefore diagonalizable by a unitary matrix. Denote the eigenvalue and the right eigenvector of 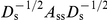 by 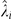 and 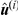, respectively. Note that 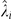 and 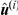 are both real and can be computed relatively easily. Then, we can obtain the relationships 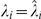, 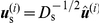, and 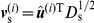. We can also obtain 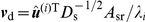 when 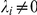.

### Example network yielding nontrivial zero eigenmodes

**Figure 5:**
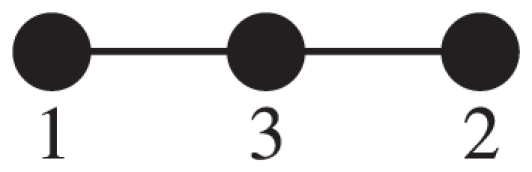
A network yielding nontrivial zero eigenvalues.

Consider the undirected network having 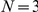 nodes as shown in Fig. 5. For this network I obtain
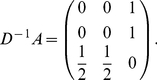
(27)By turning player 3 from GC to CD, I obtain
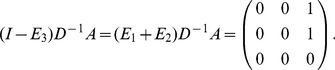
(28)All of the eigenvalues of matrix (28) are equal to zero, one trivial and two nontrivial. The one trivial zero eigenvalue originates from removing player 3 from the network of GCs. The trivial zero left eigenvector is given by 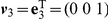. I select the two generalized zero left eigenmodes to be 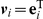 (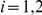). The choice of 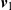 and 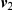 is not unique. The right eigenmodes are given by 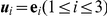.

Equation (19), for example, then reads 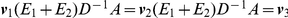 and 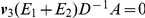.
